# Measuring Adverse Child Experiences Among Young Adolescents Globally: Relationships With Depressive Symptoms and Violence Perpetration

**DOI:** 10.1016/j.jadohealth.2019.01.020

**Published:** 2019-07

**Authors:** Robert Wm Blum, Mengmeng Li, Gia Naranjo-Rivera

**Affiliations:** Johns Hopkins Bloomberg School of Public Health, Baltimore, Maryland

**Keywords:** Adverse childhood experiences, Adolescents, Mental health, Depression, Violence, Bullying

## Abstract

**Purpose:**

The purpose of the study was to develop a measure of ACEs applicable for young adolescents in low- and middle-income countries (ACEs) and to analyze the relationships of ACEs against two outcomes: depressive symptoms and violence perpetration. There is a paucity of research on the consequences of adverse child experiences (ACEs) on adolescent health and behavior from low- and middle-income countries and virtually no multinational studies.

**Methods:**

As part of the Global Early Adolescent Study, an 11-item measure of ACEs was developed and piloted with 1,284 adolescents aged 10–14 years in 14 urban communities in an equal number of countries. With one exception where interviewers were used, data were self-reported anonymously using tablets. Results compared a summative ACEs index score and latent class analysis.

**Results:**

Findings show high rates of ACEs exposure experienced by young adolescents in resource-poor neighborhoods in low- and middle-income countries; disproportionate exposures of boys and strong associations between ACEs and both depressive symptoms and violence perpetration. Latent class analysis provided modest refinement over a summed ACEs score.

**Conclusion:**

While interventions tend to focus on behavioral outcomes, evidence suggests that ACEs exposure is a strong antecedent related to both depressive symptoms and violence perpetration.

**Implications and Contribution**

This large global study of the relationships among adverse child experiences and depressive symptoms and violence perpetration among young adolescents in resource-poor settings documents high prevalence of adversity and compares two analytic approaches that are important when developing and assessing mental health and violence prevention programs.

Research increasingly shows that adverse childhood experiences (ACEs)—a constellation of exposures including abuse, neglect, and household challenges—are linked to poor health across the life course. Specifically, having a greater number of ACEs has been associated with increased risk of many physical, mental, sexual, and behavioral health problems [1]. Although there is substantial ACEs research in the United States, global data are sparse, especially from low- and middle-income countries (LMICs). Second, few studies examine the impacts of ACEs prospectively; and fewer still assess impacts on adolescent mental health or behavior. Third, there is debate about whether the questions that comprise ACEs form a construct, index, or scale.

This study seeks to address gaps in the literature by using ACEs data from the Global Early Adolescent Study (GEAS), which examines the factors that predispose youth to sexual health risks or promote healthy sexuality. The aims of this study are (1) to present a measure of childhood ACEs developed and piloted in 15 low-income urban settings on five continents with young adolescents aged 10 to 14 years; (2) to examine the prevalence and distribution adverse exposures; and (3) to test the relative merits of analyzing ACEs both as an index with a cumulative score and as typology using latent class analysis (LCA), while exploring the association of ACEs with two outcomes: depressive symptoms and violence perpetration.

### Background

A substantial body of research shows the relationships between ACEs and long-term health consequences. Prospective studies, mostly conducted in the U.S. and Europe, demonstrate substantial associations between ACEs and poor health and life outcomes [2]. Several studies have also found a dose-response relationship between ACEs and increased risk of negative physical and behavioral health outcomes among adults in high-income countries [3], [4], including an increased risk of tobacco, alcohol, and drug abuse [4]. ACEs are associated with a higher allostatic load in midlife; and this relationship is mediated by health behaviors such as smoking, alcohol use, increased body mass index, and socioeconomic factors in early adulthood [5]. In childhood, the associations between ACEs and health status are less clear. In the global LONGSCAN Study, for example, no relationship was seen at age 12 while somatic complaints were increased at age 14 years for those who experienced 2 and more than 3 ACEs [6]. However, associations have been reported between ACEs and behavioral problems in middle childhood [7], stress and mental health in college students [8], and adult sleep disorders [9].

Fewer studies examine the health impacts of ACEs in LMICs. However, the prevalence of ACEs has been found to be higher in certain global contexts, such as 75% of respondents reporting at least one ACE in a study in the Philippines [10] compared with about 64% of respondents in the United States. Nonetheless, studies in LMICs in Africa, Asia, and Latin America have generally found similar relationships between ACEs and poor health outcomes [11], [12], [13], [14]. Cross-cultural studies on child disciplinary practices suggest that harsh and abusive discipline may be more prevalent in LMICs and that maternal age and education were key predictors for the use of punitive discipline [15].

### ACEs and Methodological Considerations

There are four prevailing approaches to measure ACEs: (1) cumulative ACE score or index, (2) weighting individual ACEs, (3) weighting ACEs by subgroup, and (4) ACEs typologies.

#### Cumulative ACEs score

The concept of ACEs was first operationalized as a construct in the ACEs study, which used the cumulative ACEs score approach [1]. Its successor, the CDC-Kaiser study, and most other ACEs studies also use the cumulative ACEs score, an index created by summing the number of unique ACEs experienced. This method does not weight responses and does not factor in frequency, duration, or intensity of exposures. This approach has consistently demonstrated an exposure-response relationship with poor health outcomes [1].

#### Weighting individual ACEs

A second approach is to weight each ACE based on certain characteristics, such as age of occurrence, frequency, duration, or perceived trauma resulting from each exposure. Events that are more recent, severe, or frequent are typically weighted more heavily. For example, Friedman et al. [16] have shown that timing, frequency, duration, and perceived severity of ACEs increased the risk of poor health outcomes.

#### Weighting ACEs by subgroup

A third approach is to derive an ACEs score by capturing the number and types of ACEs a person experienced, grouping them by category and assigning a weight to each category. These categories create a hierarchy of severity. Using this approach, an increased cumulative number of ACEs, as well as increased number of different types of ACEs, earn a higher ACEs score. Weighting by type may be important given evidence that certain adversities tend to occur in clusters and may have more detrimental health impacts [17]. The LONGSCAN Study, for example, found that clustering by type of violence exposure was significant. Specifically, the study found that witnessing domestic violence at ages 4, 6, and 8 was more associated with depression and anxiety than either physical or sexual abuse [18]. In addition, at age 12, psychological maltreatment predicted more negative outcomes than other exposures; and then at age 18, sexual abuse was the strongest predictor of negative outcomes, suggesting that different exposures have different consequences through the child life course.

#### ACEs typologies

A fourth approach is to develop typologies or determine ACEs that cluster together in statistical analyses. Using LCA, Shin et al. [19] reported that young people in a U.S. community sample could be categorized into four typologies: low ACEs (56%), household dysfunction/community violence (14%), emotional ACEs (14%), and high/multiple ACEs (16%).

The central question of this article is whether there is advantage to measuring ACEs as a cumulative index or as typologies when exploring the relationships between adversity and two adolescent outcomes: depressive symptoms and violence perpetration. In addition, if there is an advantage to using LCA to identify classes, which clusters of ACEs are most strongly associated with the outcomes of interest?

## Methodology

### Developing a measure of adversity

The measure of ACEs used in the GEAS initially consisted of 10 items (Table 1) drawn from the CDC-Kaiser ACEs Study [1], which evolved from seven ACEs initially developed by Felitti et al. [1] in their classic study of U.S. adults. The GEAS ACEs measure was modified by Kabiru et al. [11] in a study of slum-dwelling adolescents in Nairobi, Kenya.

**Table 1:** ACEs GEAS domains for statistical analysis and corresponding CDC-Kaiser study ACEs

GEAS ACEs domain	Question(s)	Corresponding CDC-Kaiser study ACEs domain^a^
Maltreatment ACEs		
Fear of being physically abused	Have you ever been scared that your parents or other adults were going to hurt you badly (so that you were injured or killed)?	Physical abuse
Fear of being emotionally abused	Have you ever been scared or felt really bad because grown-ups called you names, said mean things to you, or said they did not want you?	Emotional abuse
Physical neglect	Has there ever been a time of your life when you were totally on your own and had to take care of yourself for more than a short time?	Physical neglect
Emotional neglect	Have you ever felt like you are not loved or cared about? Have you ever felt like you have no one that protects you?	Emotional neglect
Sexual abuse	Has an adult ever touched you in your private parts except when being bathed? Has an adult ever attempted or forced you to have sexual intercourse?	Sexual abuse
Violence victimization^a^	Have you ever been bullied or threatened by boys or girls? Have you ever been slapped, hit or otherwise been physically hurt by a boy or girl in a way that you did not want?	NA—“expanded” ACE selected based on evidence in the literature
Household ACEs		
Parental substance abuse	Have your parents/guardian ever drank too much alcohol or used drugs so they came home and were really abusive to you or your family?	Household substance abuse
Parental emotional distress	Have you ever seen your mother or father so sad that they could not take care of you?	Mental illness in household
Mother treated violently	Have you ever seen your mom being hit, beaten or threatened?	Mother treated violently
Parental incarceration	Have any of your parents ever been in prison/jail?	Criminal household member
Household instability^a^	Has your family ever been forced to leave your home/house? Has there ever been a time when your family did not have enough food because they had no money?	NA—“expanded” ACE selected based on evidence in the literature

ACEs = adverse child experiences; GEAS = Global Early Adolescent Study; NA, not applicable.

a The two “expanded” ACEs, violence victimization and household instability, were included because of robust evidence in the literature that they act as adverse exposures, which was corroborated in the present analysis.

### Sampling methodology

Initially, 120 young people aged 10–14 years were identified from schools in each of the 15 country sites as part of a larger survey (Hanoi, Vietnam; Shanghai, China; New Delhi, India; Assuit, Egypt, Blantyre, Malawi, Nairobi, Kenya; Ile-Ife, Nigeria; Kinshasa, DRC; Cape Town, South Africa; Ouagadougou, Burkina Faso; Ghent; Belgium, Edinburgh, Scotland; Baltimore, MD; Cuenca, Ecuador; Cochabamba, Bolivia). For initial instrument development, the schools selected were those where the in-country investigators had previous collaborations. After obtaining parental consent and participant assent, young people completed the self-administered questions as part of a larger set of instruments (Kinshasa was the exception with interviewer administration of the measures). Subsequently, the instrument was revised and repiloted in 6 sites (Hanoi, Cuenca, Assuit, Shanghai, Blantyre, and Delhi) with a purposive sample of 75 school-going young adolescents in each site equally divided by sex and age between 10 and 14 years (www.geastudy.org). The final GEAS ACEs measure includes questions that map closely to 9 of the 10 CDC ACEs.

### ACEs measure

Eleven ACEs domains were used in the final data analysis (Table 1), eight of which had three response options—often, sometimes, never—that were coded affirmative if the response was often or sometimes. Two items used for a composite variable of violence victimization included response options as no, yes by a girl, yes by a boy, yes by both boys and girls. Responses were dichotomized yes and no. Four ACEs had multiple items that comprised the domain, and an affirmative response to any of the response options was considered to be a yes. Don't know and refuse to answer to any above item were coded as missing.

An aggregated index for ACEs exposure was created by summing the exposure to all domains with a range from 0 to 11.

### Outcomes

Analysis focused on two outcomes with robust evidence of association to ACEs: self-reported depressive symptoms [20] and violence perpetration [12].

### Depressive symptoms

A six-item depression symptom checklist was developed based on previous survey research: (1) In general, I see myself as a happy person; (2) I blame myself when things go wrong; (3) I worry for no good reason; (4) I am so unhappy I can't sleep at night; (5) I feel sad; (6) I am so unhappy I think of harming myself. Each item could be endorsed by one of five Likert scale responses: disagree a lot, disagree a little, neither disagree nor agree, agree a little, agree a lot. “Don't know” and “Refuse to answer” were recoded as missing. Responses to the first statement were inversely recoded. A summed index was generated across affirmative responses (1: agree a little, agree a lot) to five items and a negative response to the first item (1: disagree a lot, disagree a little). Outcomes ranged from 0 (no symptoms) to 6 (endorsement of all symptoms). We subsequently trichotomized responses (0, 1–3, >3 symptoms).

### Violence perpetration

Two items were used to develop a binary violence variable: (1) Have you ever bullied or threatened another boy or girl? (2) Have you ever slapped, hit or otherwise physically hurt another boy or girl in a way they did not want?

## Data Analysis

Analyses were based on the 2016–2017 piloting of the GEAS measures with 1,284 adolescents 10–14 years of age in 14 countries where there was no missing data. With the exception of sexual abuse (with 9.98% missing responses), individual items had less than 5% of responses missing. Missing values for two covariates, family wealth index (29.95%) and education attainment (2.30%), were imputed by k-nearest neighbor imputation with a k-value of 29 [21]. A two-sided *p*-value of less than .05 was considered statistically significant. Stata, version 15 (StataCorp LLC, TX), and R, version 3.3.3 (R Project), were used for analyses.

### Statistical analysis

Keeping ACEs exposure as a continuous variable, multinomial and logistic regressions were first conducted to evaluate the relationships between adversity and both depressive symptoms and violence perpetration each with and without sex stratification. LCA was then performed to explore subgroups of ACEs exposure. Further investigation was conducted to study the associations between clustered ACEs domains with each outcome, both with and without sex stratification. Regression models without sex stratification were adjusted for sex, city, family wealth index (categorized as low, medium, or high from a score calculated by principle component analysis), and educational attainment (primary vs. secondary or more). Interaction between ACEs class membership and sex was not included in the final models for both outcomes because it was found to be insignificant. Age was additionally adjusted for violence perpetration in consideration of the increased likelihood of violence engagement among older adolescents.

### Latent class analysis

**Figure 1:**
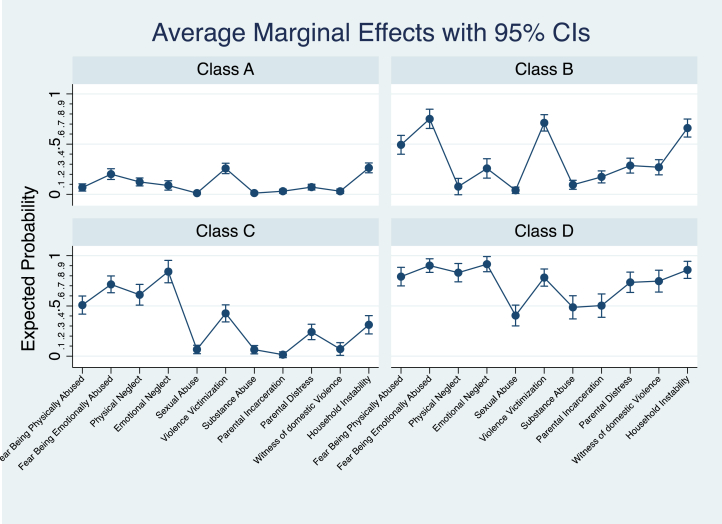
ACEs class membership profile.

Models with one to seven classes were generated, followed by model selection. Three statistical information criteria—Akaike information criterion (AIC), Bayesian information criterion (BIC), and entropy—were used to select the best model. AIC and BIC measure goodness of fit by considering the number of model parameters, and the number of parameters and observations, respectively, and entropy is a measure of model certainty. Among these criteria, BIC outperforms AIC [22]. We determined the final model when we observed an increased BIC value compared to the preceding model. A final model with a 4-class structure and entropy of .67 (fairly strong model certainty) was selected: (1) low exposure to all ACEs; (2) high fear and experience of being physically and emotionally abused, violence victimization, and household instability; (3) high fear and experience of being both physically and emotionally abused and neglected; and (4) high exposure to all ACEs (Figure 1).

The World Health Organization Ethical Review Board, the Johns Hopkins Bloomberg School of Public Health Institutional Review Board, and each site's human subjects ethical review committee approved all research protocols.

## Results

### Prevalence of adverse child experiences

As seen in Table 2, fear of being emotionally hurt (51.87%), violence victimization (45.79%), and household instability (43.07%) are the most commonly reported adversities reported by adolescents in the sample. Similarly, fear of physical violence and experiences of neglect are highly prevalent, each being reported by around one-third of the sample. Ten to 25 percent of the sample report parental incarceration, parental substance use, parental emotional distress, and witnessing their mother being treated violently; 7.17% of participants reported being sexually abused. Contrary to expectation, boys report greater exposure than girls to physical neglect (32.95% vs. 25.90%, *p* = .01), sexual abuse (8.77% vs. 5.69%, *p* = .03), violence victimization (52.27% vs. 39.82%, *p* < .01), and parental substance abuse (22.36% vs. 7.34%, *p* = .01) than girls.

**Table 2:** ACEs distribution by sex of respondents

ACEs domain	Overall (n = 1,284) (%)	Boys (n = 616) (%)	Girls (n = 668) (%)	*p*-value
Fear of being physically hurt	34.19	37.18	31.44	.03
Fear of being emotionally hurt	51.87	52.92	50.90	.47
Physical neglect	29.28	32.95	25.90	.01
Emotional neglect	38.08	38.80	27.43	.63
Sexual abuse	7.17	8.77	5.69	.03
Violence victimization	45.79	52.27	39.82	<.01
Parental substance abuse	9.27	22.36	7.34	.01
Parental incarceration	11.06	12.50	9.73	.11
Parental emotional distress	22.90	24.68	21.16	.15
Mother treated violently	17.06	17.21	16.92	.89
Household instability	43.07	41.83	42.37	.60

ACEs = adverse child experiences.

When ACEs experience was compared by sex based on class membership, distribution differences were noticed, but sex differences were nonsignificant (*p* = .07).

### Relationships between depressive symptoms and ACEs

#### ACEs as an index

Please refer to Appendix A for consistent results using different outcome specifications. The relationship between depressive symptoms and ACEs was explored two ways: first, using ACEs as a continuous index from 0 to 11 adversity exposures and second, LCA, to see if the associations with depressive symptoms were stronger for a combination of certain groups of ACEs. Overall, as ACEs exposures increase, so do the depressive symptoms independent of the type of adversity. Compared with no reported ACEs, more ACEs are positively associated with more depressive symptoms having 1, and this was true for –3 depressive symptoms (aRR: 1.23, 95% confidence interval [CI]: 1.14–1.33) and >3 symptoms (aRR: 1.70, 95% CI: 1.54–1.88). Among boys, cumulative ACEs are linked to a 22% increased risk of 1–3 depressive symptoms and a 59% increased risk of >3 symptoms (aRR: 1.22, 95% CI: 1.10–1.36 and aRR: 1.59, 95% CI: 1.38–1.82, respectively). Similar trends and slightly higher risk are observed for girls: cumulative ACEs are linked to 25% increased risk of 1–3 depressive symptoms and 88% increased risk of >3 symptoms (aRR: 1.25, 95% CI: 1.12–1.40 and aRR: 1.88, 95% CI: 1.62–2.19, respectively) (Table 3).

**Table 3:** Relationship between depressive symptoms and ACEs exposures by sex

Self-reported depressive symptoms	Overall^a^		Boys^b^		Girls^b^	
	aRR (95% CI)	*p*-value	aRR (95% CI)	*p*-value	aRR (95% CI)	*p*-value
0	Ref		Ref		Ref	
1–3	1.23 (1.14, 1.33)	<.01	1.22 (1.10, 1.36)	<.01	1.25 (1.12, 1.40)	<.01
>3	1.70 (1.54, 1.88)	<.01	1.59 (1.38, 1.82)	<.01	1.88 (1.62, 2.19)	<.01

ACEs = adverse child experiences; CI = confidence interval.

a Adjusted for sex, country, family wealth, education attainment.

b Adjusted for country, family wealth, education attainment.

#### LCA of ACEs

Analysis of depressive symptoms by class membership revealed that compared with class A (all ACEs low), adolescents with any other class of ACEs exposures have higher risk of reporting more depressive symptoms—and a stronger relationship is observed for 4 or more symptoms—with one exception discussed elsewhere in the article. Second, comparisons of outcome probabilities between any two ACEs exposure class memberships suggested that adolescents in class D (all ACEs high) have increased probability of having 4 or more depressive symptoms than those with high exposures to certain ACEs, including class B (high abuse, victimization, and instability) and class C (high abuse and neglect) with increased probabilities of 19% and 15% (both *p* < .01), respectively. In addition, those in class C (high abuse and neglect) have higher relative risk of depressive symptoms compared with those in class B (high abuse, victimization, and instability), suggesting that class C ACEs exposures, which include both abuse and neglect, may be more damaging to mental health (a decrease of 8% of having no symptoms, *p* = .02). Third, probabilities of having 1–3 or 4 or more depressive symptoms do not differ by class B or class C exposures (*p* = .35 and .27, respectively).

Stratified by sex, adolescent boys in class B are the only group without a significant relationship to depressive symptoms (1–3 symptoms: aRR: .98, 95% CI: .57–1.67; >3 symptoms: aRR: 1.93, 95% CI: .78–4.81). However, adolescent boys in class C have 2.73 and 8.36 times greater risk for 1–3 and >3 depression symptoms than those in class A, and boys in class D have a 2.88-fold and 16.09-fold increase in the risk of 1–3 and >3 symptoms. Similarly, girls with classes B, C, and D exposures have higher risks of depression. Specifically, adolescent girls in class B have 2.12 and 12.11 times greater risk for 1–3 and >3 depression symptoms than those in class A. Girls in class C have 2.76 and 14.18 times greater risk for 1–3 and >3 depression symptoms, and those in class D have a 2.97-fold and 56.74-fold increase in the risk of 1–3 and >3 symptoms (Table 4).

**Table 4:** Self-reported depressive symptoms by ACEs class membership and sex

Depressive symptoms	Boys					Girls				
	0	1–3		>3		0	1–3		>3	
		aRR (95% CI)	*p*-value	aRR (95% CI)	*p*-value		aRR (95% CI)	*p*-value	aRR (95% CI)	*p*-value
Class B (high abuse, victimization, instability)	Ref	.98 (.57, 1.67)	.93	1.93 (.78, 4.81)	.16	Ref	2.12 (1.22, 3.70)	.01	12.11 (4.82, 30.40)	<.01
Class C (high abuse and neglect)	Ref	2.73 (1.42, 5.23)	<.01	8.36 (3.50, 19.99)	<.01	Ref	2.76 (1.44, 5.30)	<.01	14.18 (5.88, 34.19)	<.01
Class D (all ACEs high)	Ref	2.88 (1.11, 7.49)	.03	16.09 (5.15, 50.25)	<.01	Ref	2.97 (1.14, 7.78)	.03	56.74 (17.44, 184.60)	<.01

ACEs = adverse child experiences; CI = confidence interval.

### Violence perpetration and ACEs

#### ACEs as an index

Regardless of sex, ACEs exposure is positively associated with violence perpetration (aOR: 1.40, 95% CI: 1.32–1.49). Adolescent boys and girls have increased odds as they are exposed to cumulative ACEs (boys: aOR: 1.46, 95% CI: 1.34–1.59; girls: aOR: 1.33, 95% CI: 1.21–1.45).

#### LCA of ACEs

Using the same four-class model with additional adjustment for age, youth with classes B, C, and D exposures are significantly more likely to be engaged in violence than their low-ACEs peers (class A). Adolescent boys and girls have an 11.96-fold and 4.32-fold increased odds of violence perpetration, respectively (aOR: 11.96, 95% CI: 6.08–23.51 and aOR: 4.32, 95% CI: 2.14–8.71). There is no difference in risk of perpetrating violence for those with class C versus class B exposures (*p* = 1.00), regardless of sex (*p* = 1.00). Class D exposure is associated with greater probability of violence perpetration compared with both class B and class C exposure among boys (increased probability: 25.25%: *p* < .01 and 32.75%, *p* < .01, respectively) but not among girls (increased probability: 4.81%, *p* = 1.00 and 2.68%, *p* = 1.00).

## Discussion

Using a sample of adolescents aged 10 to 14 years from low-income communities in 14 cities of the world (n = 1,284) from the Global Early Adolescence Study, the present study shows a high exposure to adversity including 45.79% who report violence victimization, 38.08% experiencing emotional neglect, and 29.28% reporting physical neglect. These rates are over 10% higher than the national average among adults in the U.S. [1]. This study also found that the two expanded ACEs, violence victimization (which includes bullying) and household instability, operate as ACEs exposures and may be important to include in future studies of ACEs in adolescence.

Contrary to common belief, it appears that boys consistently report greater exposure to ACEs and more fear of physical abuse and neglect than girls. Consistent with the literature, we observe that girls tend to exhibit greater internalizing behaviors, such as depression and rumination [23], [24], while boys tend to show greater externalizing behaviors, such as poor behavior regulation and aggression [20], [25].

In addition, our study confirms an exposure-response relationship between cumulative ACEs and worse health outcomes [1]. However, such a relationship alone cannot infer the effects of multiple adversities; and there is substantial evidence that adolescents are often exposed to polyvictimization [26], [27], [28]. While a growing body of literature has concluded variable effects of different classifications of ACEs, they all suggest the most worrisome outcome coming from high multiple exposures [29], [30], [31]. The present study shows that similar to findings from high-income countries, those exposed to more ACEs as children living in the poorest sections of LMIC cities have significantly increased risks of developing depressive symptoms and violence engagement as young adolescents compared with low exposure peers, regardless of sex.

Finally, in answer to the central question of the relative merits of LCA and a cumulative index of ACEs, we found that there is an association between cumulative ACEs exposures as measured by an ACEs index and negative outcomes (depressive symptoms and violence perpetration). Similarly, findings from LCA confirm previous research findings linking high multiple exposures to increased depressive symptoms; but in addition, LCA allows for a more granular understanding of relationships between outcomes and combinations of exposures. For example, we found that compared with exposure to violence victimization and household instability, high exposure to neglect and physical and emotional abuse were related to an increased likelihood of depressive symptoms. Our study suggests that high exposure to neglect, in addition to physical and emotional abuse, is linked to a greater likelihood of developing depressive symptoms than exposure to violence victimization and household instability. This finding is consistent with the study by Spinazzola et al. [32], which concluded on a national adolescent sample that psychological maltreatment (including emotional neglect) is associated with increased odds of depression. The relationship between psychological maltreatment and internalizing symptoms is also supported by other studies [33], [34], [35]. These findings are of particular salience when developing programs for young adolescents aimed at reducing violence perpetration and/or depression.

### Limitations

To our knowledge, this study is the first to investigate the association between ACEs exposure and health outcomes among early adolescents globally, treating adversity both in a cumulative and clustered manner. However, this study has limitations. First, the generalizability of our findings is limited to low-income urban settings in LMICs. Second, owing to cross-sectional nature of our data, the temporal relationship between ACEs and outcomes cannot be established. Third, depressive symptoms questions were drawn from previous survey research instruments; however, they have not been clinically validated with young adolescents in LMICs nor validated against another measure of depression. Thus, in the article, we speak of depressive symptoms rather than depression. Fourth, due to the sensitivity of ACEs questions, there may be underreporting of adverse experiences creating unaccounted-for bias.

## Conclusion

This study offers a unique multinational examination of ACEs in early adolescence across 14 communities globally. Its findings show high rates of ACEs exposure experienced by young adolescents in resource poor neighborhoods in LMIC; and similarly, it shows strong associations between ACEs and both depressive symptoms and violence perpetration. While often interventions are focused on behaviors (e.g. violence) or clinical symptoms (e.g. depression), the present research suggests the need to understand antecedent childhood exposure to adversity. We conclude that ACEs should be included routinely in behavioral research of adolescents whether in high or LMICs. The present study also suggests that research, practice, and policy efforts to address ACEs in early adolescence may be critical to reducing adolescent morbidities and to achieving the United Nations Sustainable Development Goals [36], the World Health Organization's Accelerated Action for the Health of Adolescents [37].
