# Supplementary material for: Measuring Adverse Child Experiences Among Young Adolescents Globally: Relationships With Depressive Symptoms and Violence Perpetration
Source: J Adolesc Health. 2019 Jul;65(1):86–93. doi: 10.1016/j.jadohealth.2019.01.020 (PMC6599173; doi:10.1016/j.jadohealth.2019.01.020)
Supplement: Appendix [file mmc1.docx]

Appendix A: Comparisons of the association between ACEs exposure and depressive symptoms by different outcome specifications

Table A1: Association between ACEs exposure and depressive symptoms (Overall Sample)


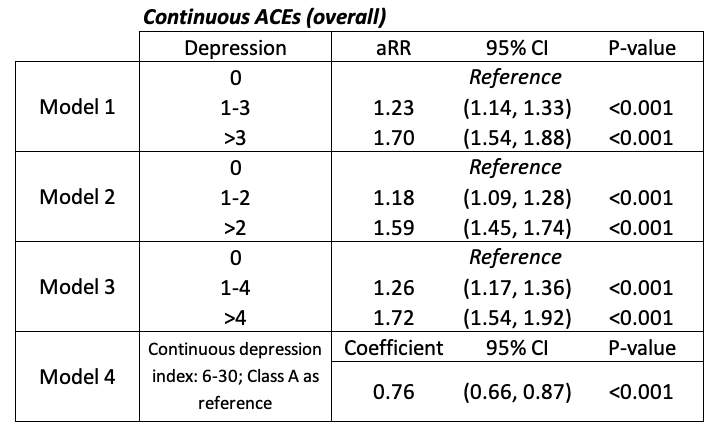


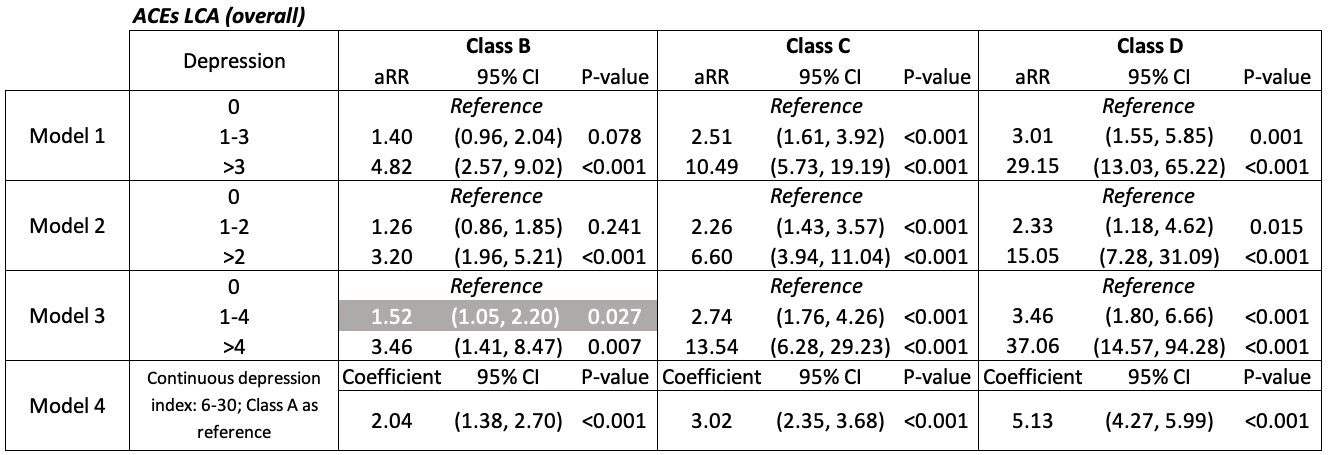


Note: Model 1 reflects the current model in the manuscript.

Table A2: Association between ACEs exposure and depressive symptoms (Among Boys Sample)


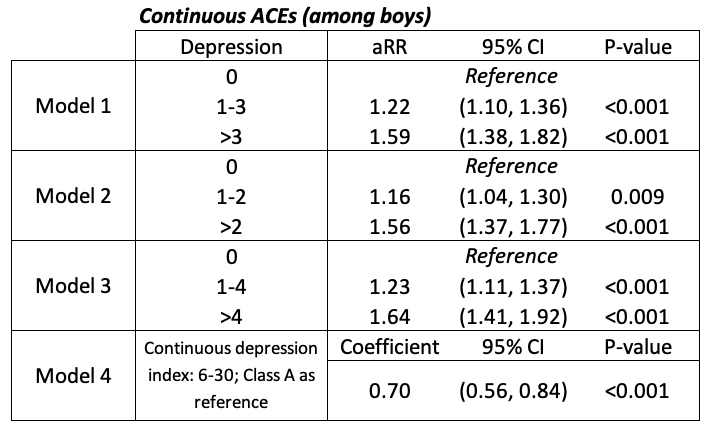


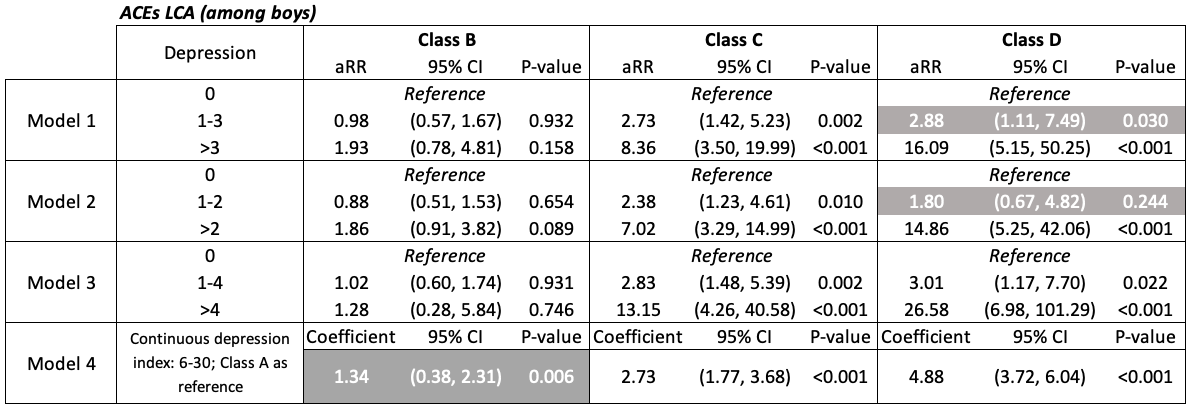


Note: Model 1 reflects the current model in the manuscript.

Table A3: Association between ACEs exposure and depressive symptoms (Among Girls Sample)


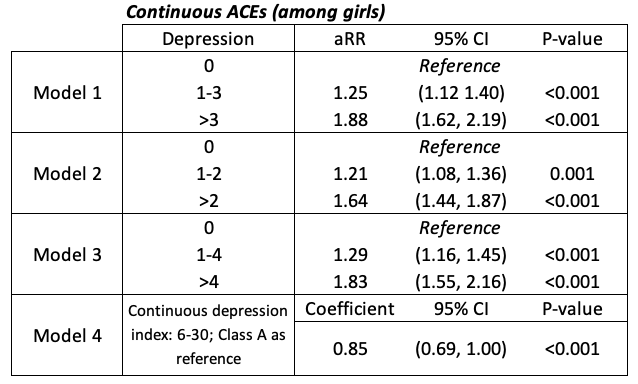


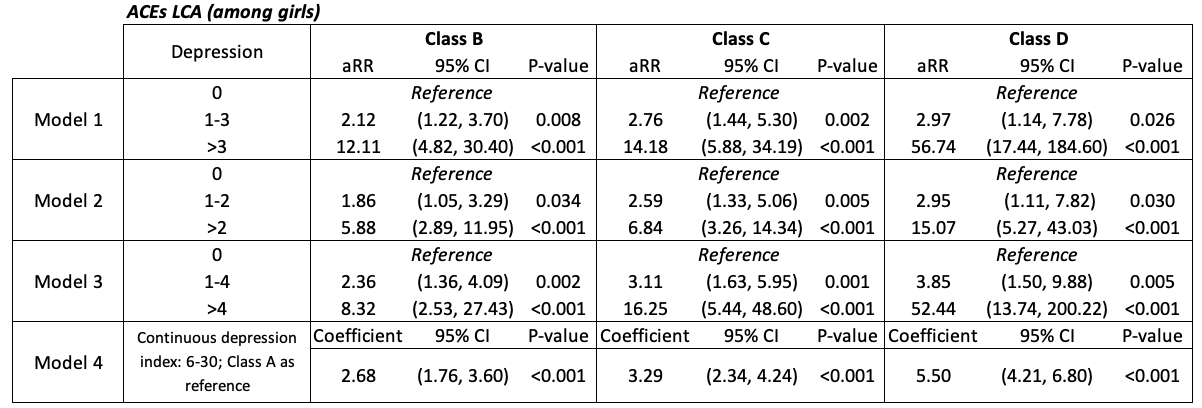


Note: Model 1 reflects the current model in the manuscript.
